# L-SAINet: A Shape-Adaptive and Inner-Scale Interaction Network for Landslide Detection in Complex Remote Sensing Scenarios

**DOI:** 10.3390/s26092812

**Published:** 2026-04-30

**Authors:** Yanchang Jia, Shuyan Hua, Hongfei Wang, Tong Jiang, Qiqi Zhao

**Affiliations:** 1 College of Geosciences and Engineering, North China University of Water Resources and Electric Power, Zhengzhou 450046, China; jiayanchang@ncwu.edu.cn (Y.J.); jiangtong@ncwu.edu.cn (T.J.); z20241110969@stu.ncwu.edu.cn (Q.Z.); 2 Yellow River Engineering Consulting Co., Ltd., Zhengzhou 450003, China; wanghf_yrec@163.com

**Keywords:** landslide detection, high-resolution remote sensing, shape-adaptive representation, channel–spatial attention, deformable feature modeling, inner-scale feature interaction, geohazard mapping

## Abstract

**Highlights:**

**What are the main findings?**

- A shape-adaptive and inner-scale interaction framework named L-SAINet is proposed for landslide detection in high-resolution optical remote sensing imagery.
- The integration of adaptive deformable convolution, channel–spatial attention, and inner-scale feature interaction improves detection accuracy, localization consistency, and robustness in complex mountain scenes.

**What are the implications of the main findings?**

- The proposed framework provides an effective solution for detecting irregular and weakly bounded landslide targets under heterogeneous background conditions.
- The shape-aware and interaction-based design can be extended to other remote sensing targets with complex geometr and strong background interference.

**Abstract:**

Landslides are widespread geohazards in mountainous regions and pose serious threats to human safety, infrastructure, and ecosystems. Accurate detection from high-resolution optical remote sensing imagery remains challenging because landslide targets often exhibit irregular morphology, large scale variation, weak boundaries, and strong background interference. To address these issues, this study proposes L-SAINet, a shape-adaptive and inner-scale interaction network for landslide detection in complex remote sensing scenarios. Built on a lightweight one-stage detection framework, the proposed method introduces an L-SAI module that integrates adaptive deformable convolution, channel–spatial attention, and inner-scale feature interaction. The shape-adaptive branch improves geometric alignment for irregular and elongated landslide bodies, while the attention branch enhances semantic discrimination under heterogeneous background conditions. The two branches are further fused at the same feature scale to construct a more unified landslide representation. Experiments on the Bijie Landslide Remote Sensing Dataset show that L-SAINet consistently outperforms the baseline detector and single-branch variants in Precision, Recall, mAP@0.5, and mAP@0.5:0.95. Additional analyses based on precision–recall curves, confusion matrices, convergence behavior, model complexity, and representative complex-scene examples further confirm its effectiveness and robustness. The results demonstrate that jointly modeling geometric adaptability and semantic refinement is an effective strategy for landslide detection in complex mountain environments.

## 1. Introduction

Landslides are among the most widespread geological hazards in mountainous regions [1,2,3,4]. They threaten human settlements, transportation infrastructure, ecological systems, and engineering activities. Rapid and reliable landslide mapping is therefore essential for hazard investigation, disaster assessment, and regional risk management [5,6,7,8,9]. In recent years, the increasing availability of high-resolution optical remote sensing imagery has provided an efficient means for large-area landslide detection. Compared with conventional field survey, remote sensing allows wider spatial coverage and shorter response time. It has become an important technical basis for landslide inventory mapping.

Despite these advantages, accurate landslide detection from optical remote sensing images remains difficult. Mountainous landscapes are highly heterogeneous. Landslides differ greatly in scale, shape, spectral response, and surface texture. Some failures appear as compact exposed patches, whereas others develop as elongated or fragmented zones along slopes, gullies, and road cuts. In many cases, their boundaries are weak and internally discontinuous. Background elements such as bare soil, terrace margins, construction disturbance, riverbank erosion, and sparse vegetation often exhibit image characteristics similar to those of landslides. Illumination variation, topographic shadow, and partial vegetation cover further reduce the separability between target and background. These factors make landslide detection fundamentally different from common object detection tasks in natural scenes [8,9].

Traditional landslide mapping methods mainly rely on manual interpretation, visual comparison, or rule-based image analysis [10,11,12,13,14]. Bitemporal inventory mapping and statistically based susceptibility modeling have also provided useful methodological background for landslide mapping and hazard assessment [15,16]. Although these approaches can be effective in local studies, they are time-consuming and strongly dependent on expert experience. With the rapid development of deep learning, convolution-based detectors have shown strong capability in remote sensing interpretation [17,18,19,20,21]. Complementary studies based on SAR/InSAR, multi-source data fusion, slope-related learning, and complex remote sensing object detection further demonstrate the importance of robust feature representation under challenging surface conditions [22,23,24,25,26,27,28,29,30,31,32,33,34]. Representative object detection frameworks have greatly improved the automation level of landslide recognition. However, most general-purpose detectors were originally designed for targets with relatively regular geometry and clearer semantic boundaries. When applied to landslide detection in mountain imagery, they often encounter two major limitations.

The first limitation lies in insufficient geometric adaptability. Landslides are non-rigid geomorphic targets. Their boundaries are commonly irregular, elongated, fragmented, or partially obscured. Standard convolution with fixed sampling geometry is not well suited to such variable spatial patterns [35,36]. As a result, predicted boxes tend to be overly loose, partially shifted, or incomplete. This weakness becomes more evident when the detector is evaluated under strict overlap thresholds, where accurate geometric alignment is required [24,25,27].

The second limitation lies in inadequate discrimination under complex background conditions. Many mountain surface units share similar local texture or spectral appearance with landslides [23,24,27,28]. A detector that lacks sufficient semantic refinement may respond to roadside excavation, bare slopes, or exposed terrace surfaces in the same way as it responds to true landslides. In such cases, improvements in target saliency alone are often insufficient if the model does not simultaneously maintain reliable spatial representation [32,33,34].

Recent studies have attempted to improve remote sensing object detection by introducing attention mechanisms, feature fusion strategies, deformable modeling, and contextual enhancement modules [37,38,39,40,41,42]. These developments have improved feature discrimination or geometric flexibility to some extent. However, for landslide detection in complex mountain scenes, two limitations remain evident. First, many existing detectors still rely on fixed or weakly adaptive spatial sampling, which makes them less effective for elongated, fragmented, and weakly bounded landslide targets [27]. Second, semantic refinement and geometric adaptation are often introduced as separate enhancements, while their interaction within the same feature scale remains limited. For landslide targets, this separation may weaken the network’s ability to jointly characterize target extent and background distinction.

To address these issues, this study proposes L-SAINet, a landslide detection framework designed for high-resolution optical remote sensing imagery in complex mountainous environments. The framework is built on a lightweight one-stage detector and introduces a Landslide Shape-Aware and Inner-scale interaction (L-SAI) module. The proposed design combines a shape-adaptive branch, a channel–spatial attention branch, and an inner-scale feature interaction mechanism. The shape-adaptive branch improves geometric alignment for irregular and elongated landslides. The attention branch enhances semantic saliency and suppresses confusing background response. The interaction mechanism enables effective collaboration between geometry-oriented and semantics-oriented features at the same feature scale, thereby improving both detection reliability and localization consistency.

It should be noted that the contribution of this study does not lie in introducing entirely new basic operators, but in constructing a task-oriented coupling design for landslide detection, where geometry-sensitive and semantics-sensitive enhancements are jointly organized for irregular geomorphic targets in complex mountain scenes.

The main contributions of this study are summarized as follows:

(1) A task-oriented landslide detection framework is developed for high-resolution optical remote sensing imagery in complex mountainous scenes.
(2) A shape-aware feature enhancement module is designed to jointly model geometric adaptation and semantic refinement through inner-scale feature interaction.
(3) Extensive experiments on the Bijie Landslide Remote Sensing Dataset demonstrate that the proposed framework improves landslide detection performance, especially under strict localization criteria and complex background conditions.

## 2. Data and Methodology

### 2.1. Dataset Description and Task Definition

This study used the publicly available Bijie Landslide Remote Sensing Dataset released by Wuhan University as the experimental data source. The images used in this work were directly obtained from the public dataset rather than newly cropped from original large-scene remote sensing imagery in this study. The dataset was organized into training, validation, and test subsets at a ratio of 80%, 10%, and 10%, corresponding to 3514, 439, and 439 image patches, respectively. The public annotations provided with the dataset were adopted for single-class landslide detection, and the labels were manually checked against the image content before model training to verify consistency between the released annotations and visible landslide targets.

The average target size of approximately 118 × 162 pixels indicates that the landslide instances in the dataset generally occupy a moderate spatial extent in the image patches rather than appearing as extremely tiny objects. However, because landslides in mountainous environments often exhibit irregular outlines, elongated extension patterns, and fragmented morphology, this average value should not be interpreted as implying uniform target geometry. Instead, it suggests that the detector must simultaneously preserve sufficient semantic discrimination and geometric adaptability for landslide targets with variable extent and boundary complexity.

Axis-aligned bounding boxes were adopted in this study because the primary objective was rapid landslide target detection rather than fine-grained boundary delineation. Compared with rotated boxes or polygon annotations, horizontal boxes are easier to annotate consistently at large scale and are more suitable for efficient detector training and evaluation. However, this representation cannot fully describe the detailed geometry of highly elongated or irregular landslide bodies, which also constitutes a limitation of the current work.

The main statistical characteristics of the dataset are summarized in Table 1.

The statistical properties of the annotated boxes further reflect the geometric complexity of the targets. Landslides do not show a fixed clustering pattern in image space, and their sizes and aspect ratios vary substantially. These characteristics indicate pronounced geometric heterogeneity. Under such conditions, conventional detectors with fixed receptive fields often struggle to represent landslide boundaries in a stable manner, especially for elongated failures, fragmented targets, and landslides embedded in heavily disturbed backgrounds. These dataset characteristics provide the direct motivation for the shape-adaptive design proposed in this study [24,35,36,41].

Based on these considerations, the research task was defined as single-class landslide detection in high-resolution optical remote sensing imagery. The goal was not only to improve detection rate, but also to maintain reliable localization consistency under complex background conditions. Particular attention was given to model behavior at high intersection over union thresholds. Unlike common natural scene objects, landslides rarely have sharp and closed boundaries. Their outlines are jointly controlled by topography, lithology, and disturbance processes. A useful detector must therefore possess both semantic discrimination and geometric adaptation in order to meet the practical needs of landslide inventory mapping and hazard investigation.

### 2.2. Overall Architecture of L-SAINet

To address the irregular geometry, large scale variation, and strong background interference of landslide targets, this study developed the L-SAINet detection framework. The model was implemented based on a lightweight one-stage detector and retained the standard backbone–neck–head paradigm. The backbone was responsible for hierarchical feature extraction, the neck aggregated and transmitted multi-scale features, and the decoupled detection head separately performed classification and bounding-box regression.

**Figure 1:**
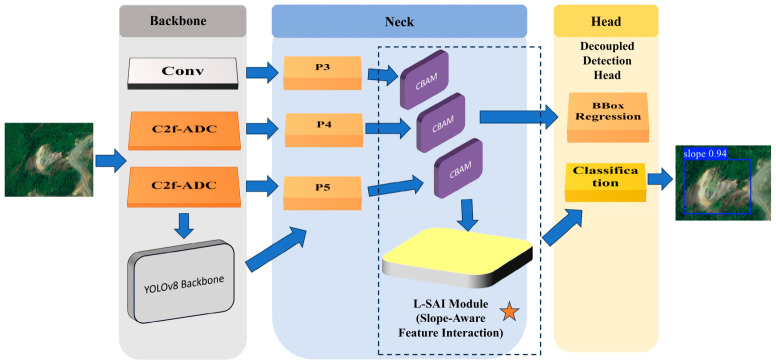
Overall architecture of the proposed L-SAINet framework.

As shown in Figure 1, the key modification of L-SAINet is the introduction of task-oriented feature enhancement components into the detector pipeline for multi-scale representation learning. Specifically, CBAM-based semantic refinement is applied to relatively shallow and intermediate stages, while ADC-based shape-adaptive modeling is introduced in deeper stages to better capture irregular landslide geometry. The detector retains a standard three-scale prediction head on P3/8, P4/16, and P5/32 feature maps. Through this design, geometric adaptation and semantic refinement are jointly incorporated without changing the basic one-stage detection paradigm.

Compared with a general-purpose detector, the main improvement of L-SAINet does not lie in increasing network depth or parameter volume, but in constructing a task-oriented feature learning strategy that jointly considers geometric adaptability and semantic refinement for landslide targets. This design is intended to improve robustness under heterogeneous backgrounds while preserving satisfactory localization quality for irregular and weakly bounded landslides.

### 2.3. L-SAI Module Design

#### 2.3.1. Shape-Adaptive Branch

In optical remote sensing images, landslides often appear as elongated, curved, fragmented, or weakly bounded patches. Their morphological characteristics differ substantially from those of regular objects in natural scenes. Fixed receptive fields are more suitable for compact targets, but they have limited ability to capture the boundaries of non-rigid geomorphic units. This weakness becomes more evident when slope shadow, vegetation cover, and engineering disturbance occur simultaneously. Under such conditions, feature responses tend to spread or shift away from the true target. To address this issue, a shape-adaptive branch was introduced into the proposed detector for geometric feature refinement. This branch uses adaptive deformable convolution to dynamically adjust sampling locations and thus strengthen the representation of geometric heterogeneity.

(1) $$y\left(p_{0}\right)=\sum_{p_{n}\in R}w\left(p_{n}\right)\cdot x\left(p_{0}+p_{n}+\Delta p_{n}\right)$$

where $p_{0}$ denotes the reference location on the feature map, R represents the regular sampling grid, $w(p_{n})$ is the convolution weight, and $\Delta p_{n}$ denotes the learned offset for adaptive sampling. This formulation enables the receptive field to better align with irregular and elongated landslide structures.

**Figure 2:**
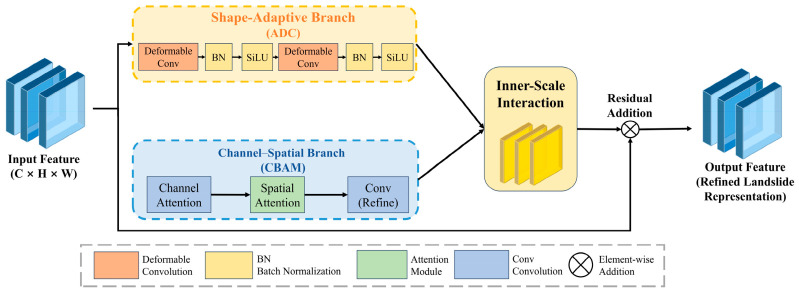
Structure of the proposed L-SAI module. The input feature is processed in parallel by a shape-adaptive branch and a channel–spatial branch. Their outputs are integrated through an inner-scale interaction operation, and the final enhanced feature is obtained through residual addition to generate a refined landslide representation.

By learning spatial offsets from the input feature map, the branch reconstructs the convolutional sampling pattern so that the receptive region can better align with landslide boundaries and extension directions. Compared with fixed convolution, this mechanism provides more effective geometric alignment for irregular patches, elongated landslides, and locally fragmented failures. It helps reduce excessive enclosure and partial omission in predicted boxes. In the proposed framework, this branch operates on the enhanced feature extraction pathway so that geometric differences can be perceived and refined before the final prediction stage. Its central role is not simply to amplify response intensity, but to improve the geometric form of intermediate representation and provide more stable spatial support for subsequent box regression in Figure 2.

In the present study, “shape-adaptive” does not simply refer to the use of deformable convolution as an isolated operator. Rather, it refers to the ability of the detector to adjust spatial sampling in response to the irregular, elongated, and fragmented geometric patterns of landslide targets, thereby improving the consistency between intermediate feature representation and target extent.

#### 2.3.2. Channel-Spatial Attention Branch

Besides geometric complexity, landslide detection is also challenged by strong background confusion. Bare soil, terrace edges, road cuts, riverbank erosion zones, and gaps within forest cover often resemble landslides in local roughness, tone, or structure. At the same time, illumination variation, shadow cover, and vegetation masking further weaken the contrast between target and background. To improve discriminative capability, a joint channel and spatial attention branch was constructed within the L-SAI module [32,33,34].

(2) $$F^{\prime }=M_{c}\left(F\right)⊗F$$

(3) $$F^{\prime \prime }=M_{s}\left(F^{\prime }\right)⊗F^{\prime }$$

where F denotes the input feature, $M_{c}\left(\cdot \right)$ and $M_{s}(\cdot )$ denote the channel attention and spatial attention operations, respectively, and ⊗ represents element-wise multiplication. Through this sequential refinement, the network enhances landslide-related responses while suppressing confusing background information.

This branch first evaluates the importance of feature responses along the channel dimension. It strengthens those channels that are related to landslide morphology, exposed ground, and disturbed boundaries. It then highlights potentially relevant regions in the spatial dimension and suppresses responses unrelated to the detection task. Through this two-stage refinement process, the network can focus more stably on landslide bodies in complex mountain scenes and reduce false responses caused by vegetation cover, dark shadow, and disturbed anthropogenic ground. Compared with a strategy that relies only on geometric deformation, the attention branch emphasizes semantic distinction among surface units and serves as a critical complement for reliable landslide recognition.

#### 2.3.3. Inner-Scale Feature Interaction

Most existing detection frameworks emphasize information transfer across scales, but make limited use of interaction among different feature types within the same scale. For landslide detection, if shape-adaptive information and semantic enhancement are propagated independently, it is difficult to form a unified representation of target extent and background distinction. To address this issue, an inner-scale feature interaction mechanism was introduced into the L-SAI module. The outputs of the shape-adaptive branch and the attention branch were first aligned at the same spatial resolution and then fused to generate an enhanced feature representation.

Here, “inner-scale interaction” specifically refers to feature interaction between different enhancement branches at the same pyramid level, rather than information aggregation across different feature scales.

(4) $$F_{sa}=\phi_{adc}\left(F\right),F_{att}=\phi_{att}\left(F\right)$$

(5) $$F_{out}=F+\phi_{fus}\left(F_{sa},F_{att}\right)$$

where F denotes the input feature, $F_{sa}$ and $F_{att}$ represent the outputs of the shape-adaptive branch and the attention branch, respectively, $\phi_{fus}\left(\cdot \right)$ denotes the inner-scale fusion operation, and $F_{out}$ is the final enhanced feature. The residual connection preserves the original geomorphic information and improves training stability.

The key idea is that geometric representation and semantic representation should not be treated as isolated responses. Instead, they should interact under a consistent feature scale so that boundary-sensitive information can be constrained by semantic saliency, while semantically focused regions can obtain more reliable spatial support. Through this design, the L-SAI module forms a more unified intermediate representation for landslide detection rather than a simple stacking of independent enhancement components.

Unlike conventional cross-scale fusion, which mainly aggregates features from different pyramid levels, the proposed inner-scale interaction is designed to couple geometry-oriented and semantics-oriented responses within the same feature scale, so that target extent modeling and background discrimination can be jointly refined before prediction.

### 2.4. Optimization Objective

A decoupled strategy was adopted in the detection head so that classification and localization could be learned along separate paths. This design reduces mutual interference between the two objectives during optimization and allows category confidence and box regression to adapt to their own feature distributions.

The overall training objective for single-class landslide detection consisted of classification loss, bounding-box regression loss, and distribution focal loss:

(6) $$L=\lambda_{cls}L_{cls}+\lambda_{box}L_{box}+\lambda_{dfl}L_{df}l$$

where $L_{cls}$ denotes the classification loss, $L_{box}$ denotes the bounding-box regression loss, and $L_{dfl}$ denotes the distribution focal loss. The corresponding balancing coefficients were set to $\lambda_{cls}$ = 0.5, $\lambda_{box}$ = 7.5, and $\lambda_{dfl}$ = 1.5, following the standard optimization setting of the baseline detector. All compared models were trained with the same loss configuration to ensure fair comparison.

The classification term constrains the confidence of predicted boxes for landslide presence, while the localization terms focus on spatial overlap and geometric consistency between predicted boxes and reference annotations. Since this study emphasizes reliable localization under complex background conditions, the model was required not only to identify landslide targets correctly, but also to place predicted boxes as close as possible to the actual target extent.

## 3. Experimental Design

### 3.1. Implementation Details

To ensure a fair comparison among all models, the baseline detector, the single-branch enhanced variants, and the complete L-SAINet were trained and evaluated using the same dataset split and the same experimental pipeline. All experiments were conducted for 200 epochs with a batch size of 16 and an input image size of 640 × 640. The training process used pretrained weights, automatic optimizer selection, and mixed-precision training. The random seed was fixed to 0, and deterministic training was enabled to improve reproducibility.

The initial learning rate was set to 0.01, with a final learning rate factor of 0.01. The momentum and weight decay were set to 0.937 and 0.0005, respectively. A warm-up strategy was adopted, with 3 warm-up epochs, warm-up momentum of 0.8, and warm-up bias learning rate of 0.1.

Data augmentation mainly included HSV perturbation (h = 0.015, s = 0.7, v = 0.4), translation (0.1), scaling (0.5), horizontal flipping (0.5), and mosaic augmentation. Mosaic augmentation was closed in the final 10 epochs. Rotation, shear, perspective transformation, MixUp, and CutMix were not used in the present training setup.

Model selection was based on validation-set performance, and the final results were reported on the independent test set. All compared models were trained under the same optimization settings so that the observed performance differences could be attributed mainly to architectural variation rather than to inconsistent training conditions.

### 3.2. Evaluation Metrics

Precision, Recall, mAP at an intersection over union threshold of 0.5, and mAP averaged from 0.5 to 0.95 were adopted as the main evaluation metrics. Precision reflects the reliability of detected results identified as landslides. Recall measures the ability of the model to cover true landslide targets. mAP at 0.5 evaluates overall detection performance under a relatively relaxed threshold. mAP from 0.5 to 0.95 places stronger emphasis on localization quality and box consistency across increasingly strict overlap conditions.

For landslide detection, detection rate under a low overlap threshold alone is not sufficient to demonstrate practical utility. Landslide targets commonly have irregular boundaries and internally heterogeneous texture. If a predicted box encloses a much broader area than the actual target, it may still be counted as correct under a relaxed threshold, yet such a result is of limited value for hazard inventory mapping and risk assessment. For this reason, mAP from 0.5 to 0.95 was treated in this study as a key indicator of geometric representation capability, and special attention was given to performance differences under this metric.

In addition, normalized confusion matrices and precision-recall curves were used to provide auxiliary analysis of classification stability. The confusion matrix was used to examine the degree of confusion between the landslide class and background. The precision-recall curve was used to inspect the balance between detection reliability and target coverage under different confidence thresholds. This multi-metric evaluation framework enables a more comprehensive assessment of model behavior in complex mountain scenes.

### 3.3. Comparative Settings

To clarify the contribution of each key component, four model configurations were compared. The first was the baseline detector, which did not include any task-oriented enhancement. The second added the channel and spatial attention module to evaluate the contribution of semantic refinement under complex background conditions. The third added the shape-adaptive module to assess the effect of geometric modeling on landslide boundary representation. The fourth was the complete L-SAINet, which combined the attention branch, the shape-adaptive branch, and the inner-scale feature interaction mechanism to evaluate their collaborative effect.

The purpose of this comparison was not merely to show that the complete model achieved a higher score. Rather, it was to trace which type of problem was improved by each enhancement path. The attention branch mainly addressed background suppression and semantic discrimination. The shape-adaptive branch primarily improved boundary alignment and representation of irregular targets. The inner-scale interaction mechanism integrated these two streams into a unified feature expression. With this design, the conclusions of the study can be built upon explicit structural differences instead of abstract performance comparison alone.

## 4. Results

### 4.1. Overall Detection Performance

In addition to internal ablation variants, representative lightweight and two-stage detectors were also compared to assess the competitiveness of L-SAINet against alternative detection frameworks. As shown in Table 2, L-SAINet achieved the highest Precision, Recall, mAP@0.5, and mAP@0.5:0.95 among all compared models, while maintaining acceptable computational cost. Compared with YOLOv8m, YOLOv10n, YOLO11n, and Faster R-CNN, the proposed model showed more balanced gains in both detection reliability and localization quality, indicating that the task-oriented design is effective not only relative to the baseline detector but also relative to other representative detection frameworks.

To further clarify the contribution of each proposed component, internal ablation results are reported in Table 3. The complete L-SAINet achieved the best performance among all variants, indicating that semantic refinement and shape-adaptive modeling become more effective when combined through inner-scale interaction.

**Figure 3:**
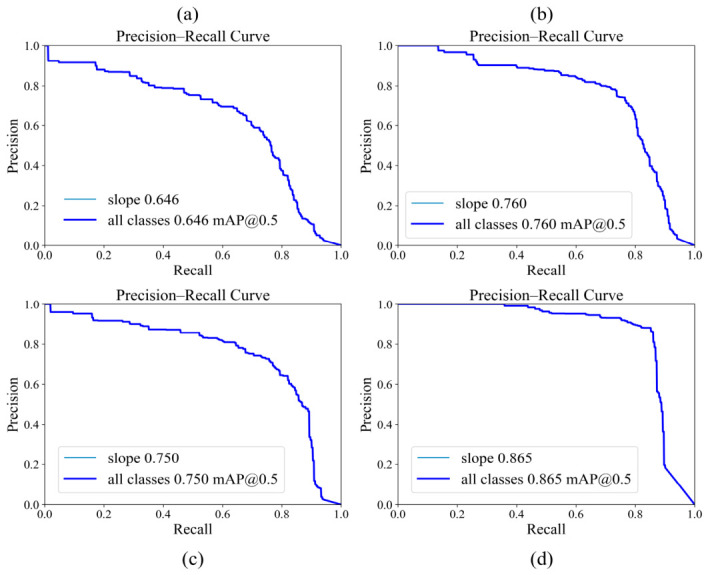
Precision–recall curves of different model variants. (**a**) YOLOv8m; (**b**) YOLOv8m + CBAM; (**c**) YOLOv8m + ADC; (**d**) L-SAINet.

Figure 3 further illustrates the overall detection quality. The precision–recall curves show that L-SAINet maintains a high level of precision across a broad confidence range. This suggests that the model is able to expand target coverage without markedly sacrificing result reliability. The normalized confusion matrices indicate a clear increase in correct identification of the landslide class and a corresponding reduction in background confusion. Together, To further clarify the contribution of each proposed component, internal ablation results are reported in Table 3. The complete L-SAINet achieved the best performance among all variants, indicating that semantic refinement and shape-adaptive modeling become more effective when combined through inner-scale interaction.

**Figure 4:**
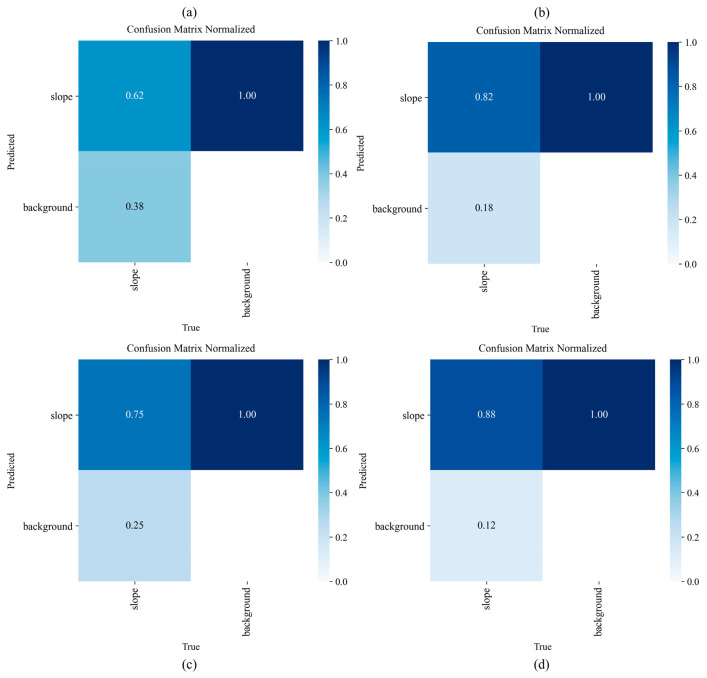
Normalized confusion matrices of different model variants. (**a**) YOLOv8m; (**b**) YOLOv8m + CBAM; (**c**) YOLOv8m + ADC; (**d**) L-SAINet.

Table 3, Figure 3 and Figure 4 show that the advantage of L-SAINet is not confined to a single evaluation metric, but is reflected in a coordinated improvement in overall detection behavior.

It is important to note that the observed performance gain should not be interpreted only in terms of numerical increase. Improvement in mAP at 0.5 indicates enhanced ability to detect landslide targets. The marked gain in mAP from 0.5 to 0.95 further suggests that the model can preserve satisfactory localization quality under strict overlap requirements. This pattern is highly consistent with the structural design proposed in this study. Shape adaptation improves spatial alignment, semantic enhancement reduces background confusion, and inner-scale interaction allows the two forms of information to support each other within a unified representation.

### 4.2. Training Convergence Analysis

**Figure 5:**
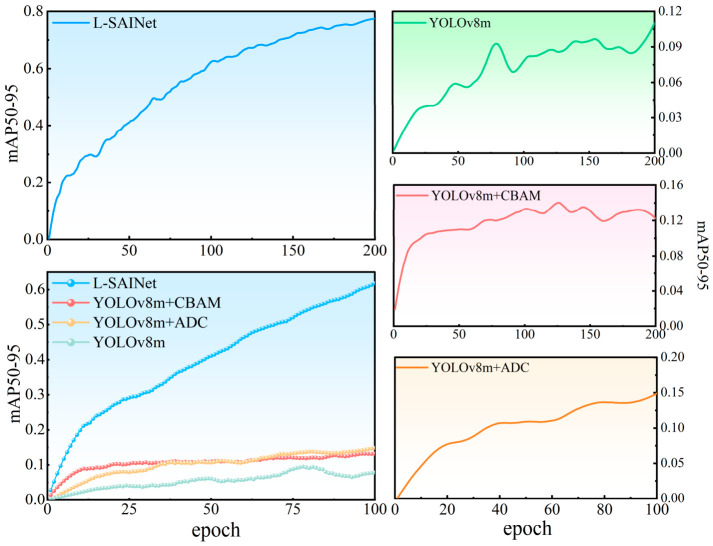
Training convergence curves of different model variants in terms of mAP@0.5 and mAP@0.5:0.95. The left panels show the overall convergence trends of the proposed model and all compared variants, while the right panels present the mAP@0.5:0.95 evolution of individual baseline configurations during training.

Figure 5 presents the performance evolution of different model variants during training. In terms of mAP@0.5, the complete model shows a rapid increase in the early training stage and maintains a clear lead throughout subsequent iterations. This pattern indicates that the model can establish an effective representation of landslide targets at an early stage. For mAP@0.5:0.95, the growth trend of L-SAINet is even more pronounced, and the curve continues to rise steadily in the middle and late stages of training. This suggests that the model has stronger optimization potential in precise boundary localization.

The recorded training logs further support the stability of the optimization process. In the later training stage, the validation performance remained relatively stable at a high level, while the training box loss, classification loss, and distribution focal loss decreased from about 1.370, 0.915, and 1.748 to 0.635, 0.348, and 1.145, respectively. These results indicate that the proposed framework maintained stable convergence behavior and continued to improve its localization capability during the later training epochs.

By contrast, models with only the attention branch or only the shape-adaptive branch show some improvement, but the magnitude remains limited and does not approach the convergence level of the full model. The superior convergence speed and late-stage stability of L-SAINet suggest that its advantage does not emerge only at the final test stage, but is gradually accumulated throughout the optimization process.

**Figure 6:**
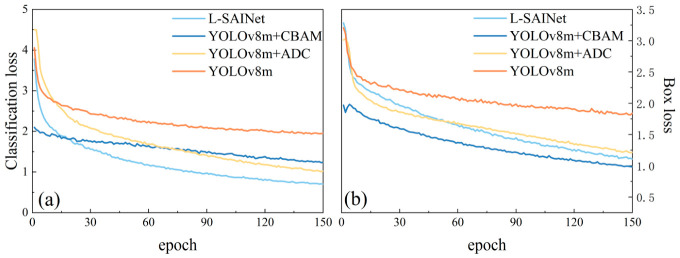
Convergence comparison of different model variants during training. (**a**) Classification loss; (**b**) box loss. The proposed L-SAINet shows a more stable optimization trend and lower final loss values than the compared variants.

The evolution of precision also shows that the complete model fluctuates less during training. This indicates that the introduction of inner-scale feature interaction creates a more stable constraint between geometric response and semantic response. Such stability is especially important in mountain scenes with complex backgrounds, where a detector that relies excessively on local texture often produces inconsistent responses across samples. The superior convergence speed and stability of L-SAINet suggest that its advantage does not emerge only at the final test stage, but is gradually accumulated throughout the whole optimization process in Figure 6.

### 4.3. Ablation Study

To analyze the role of each key component, ablation experiments were conducted, and the results are listed in Table 4. The baseline model, YOLOv8m, achieved a Precision of 0.69, a Recall of 0.62, an mAP@0.5 of 0.646, and an mAP@0.5:0.95 of 0.104. After adding only the attention module, the corresponding values increased to 0.78, 0.82, 0.760, and 0.129, respectively. After adding only the shape-adaptive module, the model achieved a Precision of 0.74, a Recall of 0.75, an mAP@0.5 of 0.750, and an mAP@0.5:0.95 of 0.148. After combining CBAM and ADC without inner-scale interaction, the performance further increased to a Precision of 0.80, a Recall of 0.83, an mAP@0.5 of 0.792, and an mAP@0.5:0.95 of 0.228. When inner-scale interaction was further introduced, the complete L-SAINet achieved the best performance, with a Precision of 0.86, a Recall of 0.88, an mAP@0.5 of 0.865, and an mAP@0.5:0.95 of 0.612.

These results indicate that the attention mechanism and the shape-adaptive mechanism improve detection in different ways. The main contribution of the attention branch lies in enhancing the saliency of landslide regions and suppressing confusion caused by vegetation, shadow, and disturbed ground. It is therefore helpful for improving detection rate and general precision under relaxed evaluation thresholds. However, this branch does not directly constrain the geometric tightness of the predicted box. As a result, better semantic focus does not necessarily lead to better localization under strict overlap conditions. This indicates that the attention branch improves detection reliability under relaxed and moderately strict criteria, but its contribution to high-precision localization remains limited when used alone.

**Figure 7:**
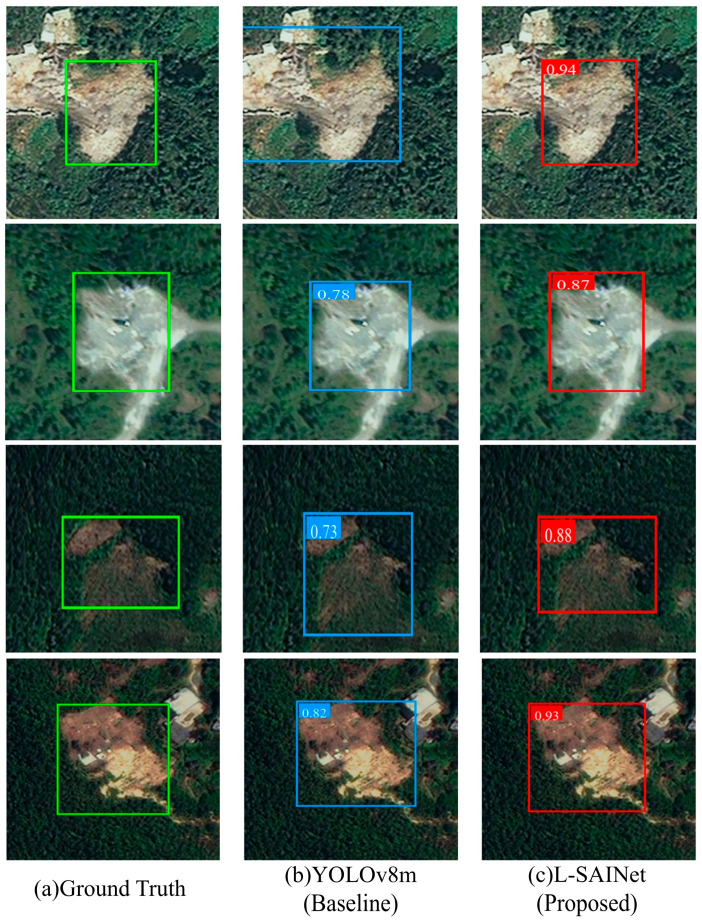
Representative qualitative comparison among (**a**) ground-truth annotations, (**b**) baseline predictions, and (**c**) L-SAINet predictions.

By comparison, the shape-adaptive branch acts more directly on spatial sampling and thus improves the geometric alignment of elongated and weakly bounded landslides. Its influence is more evident in localization quality under stricter thresholds. Nevertheless, when it is used alone, its ability to suppress confusing backgrounds remains limited, and its main strength lies in boundary representation rather than semantic filtering. The significant gain achieved by the complete model should therefore not be interpreted as a simple sum of module effects. Rather, it indicates that semantic enhancement and geometric modeling become substantially more effective when they interact within the same scale. The visual comparison shown in Figure 7 supports this interpretation. Compared with the baseline and the single-branch variants, L-SAINet produces boxes that are more consistent with the true spatial extent of elongated, fragmented, and complex-background landslides.

In addition to detection accuracy, the computational cost of each configuration was also evaluated. As summarized in Table 5, L-SAINet introduces a moderate increase in parameters and floating-point operations compared with the baseline detector, while still maintaining real-time inference capability.

### 4.4. Performance Under Complex Scene Conditions

These representative scene categories were manually grouped according to dominant visual and geomorphic characteristics in the test set, including shadow influence, vegetation cover, road-cut disturbance, multi-scale target coexistence, and complex textured background.

**Figure 8:**
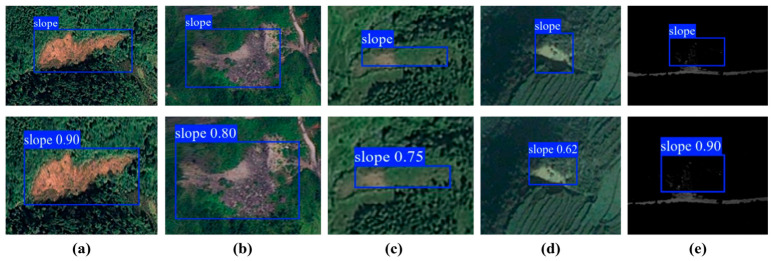
Detection results under representative complex scene conditions. (**a**) Shadow-affected low-contrast scene; (**b**) vegetation-covered slope; (**c**) road-cut disturbance scene; (**d**) small landslide near linear infrastructure; (**e**) complex background scene.

The practical difficulty of landslide detection in remote sensing imagery is mainly concentrated in low-contrast regions, vegetation-covered slopes, road-cut disturbance zones, multi-scale scenes, and areas with complex texture. Figure 8 presents representative detection results of L-SAINet under these typical conditions. In regions where shadow cover reduces the brightness of the target, the model still captures the landslide extent in a relatively stable manner. Under vegetation masking, where the target boundary is not clearly expressed, the predicted box still maintains a reasonable enclosure relationship. For landslides associated with road construction and slope cutting, the model can also isolate valid target response from a highly disturbed background. The quantitative performance under representative difficult scene categories is summarized in Table 6.

In scenes where landslides of different sizes occur together, the model shows satisfactory adaptability to large, medium, and small targets. For irregular landslides embedded in highly textured backgrounds, the predicted boxes still remain well aligned with the true target extent. These observations indicate that L-SAINet does not perform well only on relatively ideal samples. It also preserves a certain level of stability under the complex surface conditions commonly encountered in mountain remote sensing images. Combined with the ablation results discussed above, this robustness can be attributed to the simultaneous enhancement of geometric representation and semantic discrimination, which allows the network to maintain reliable detection behavior under mixed background and variable morphology.

### 4.5. Large-Sample Qualitative Evaluation

**Figure 9:**
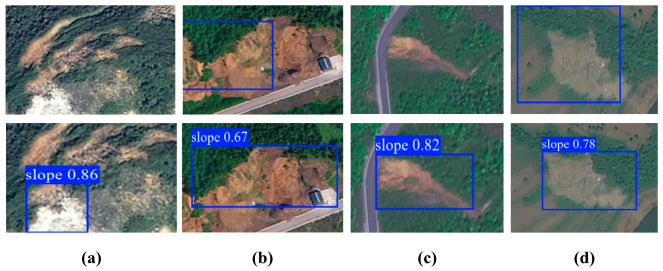
Additional qualitative examples in complex mountain scenes. (**a**–**d**) Representative landslide detection cases under complex mountain backgrounds.

Figure 9 shows detection results over a larger set of test samples in order to inspect the overall localization behavior of the model. In most examples, L-SAINet can correctly identify landslide regions and produce high-confidence bounding boxes with concentrated spatial response. For the targets that are successfully detected, the predicted positions show strong agreement with the actual target extent. This suggests that the model provides relatively good localization accuracy and geometric consistency during inference.

At the same time, a small number of missed detections still appear under extremely complex conditions, especially where background texture is highly confusing, local occlusion is severe, or the contrast between landslide and surrounding ground is very weak. The examples shown in Figure 9 suggest that the current framework performs well in providing reliable spatial constraints for detected targets, but complete coverage of all difficult cases remains limited by image conditions and target separability. In other words, L-SAINet is more effective at producing accurate localization for detected landslides than at fully eliminating omission in all challenging situations. This observation is consistent with the practical characteristics of landslide recognition in complex mountain terrain and also points to the potential value of future improvements based on multi-source data or temporal constraints.

## 5. Discussion

### 5.1. Effect of Shape-Adaptive Modeling

The experimental results indicate that shape-adaptive modeling plays an important role in landslide detection [24,25,35,36,41]. This finding is closely related to the geomorphic nature of the target itself. Unlike compact man-made objects, landslides usually have irregular outlines and highly variable elongation patterns. Their visible extent is controlled by slope morphology, material accumulation, vegetation cover, and anthropogenic disturbance. Under such conditions, fixed geometric sampling is often unable to maintain stable boundary alignment. This limitation directly affects the quality of box regression.

Accordingly, the term “shape-adaptive” in this work is used in a task-specific sense, emphasizing adaptive spatial support for irregular geomorphic targets rather than claiming a complete geometric model of landslide shape.

The performance of the model variant with the shape-adaptive branch supports this interpretation. Compared with the baseline detector, the ADC-enhanced variant produced better localization behavior and achieved slightly higher performance under stricter overlap thresholds. Although the gain was still limited when the module was used alone, the trend suggests that spatially adaptive sampling contributes to more accurate representation of elongated and weakly bounded landslide targets. This effect is especially meaningful for remote sensing scenes where geometric complexity rather than spectral contrast becomes the main source of uncertainty.

Another important implication is that landslide detection should not be treated as a purely semantic recognition problem. In practice, many errors originate not from a complete failure to identify the target, but from an inability to fit the actual target extent. From the perspective of hazard inventory mapping, a detector with weak geometric consistency may provide visually acceptable results under relaxed thresholds, yet its practical value remains limited. The observed improvement in high-threshold performance therefore confirms the necessity of introducing shape-aware modeling into the detection framework [24,27,35,36].

### 5.2. Role of Attention-Based Feature Refinement

The attention branch mainly improves feature discrimination under complex background conditions [28,32,33,34]. This contribution is particularly important in mountainous optical imagery, where non-landslide surfaces frequently resemble landslide targets in local texture and color. Bare soil, disturbed road cuts, terrace margins, and shadow-covered slopes often produce misleading responses. Without semantic refinement, the detector can easily assign high confidence to visually similar but geologically irrelevant regions.

The ablation results show that the attention module improves detection performance under relatively relaxed evaluation conditions. This indicates that it helps the network focus on potentially relevant landslide areas and suppress irrelevant background response. In other words, the attention branch strengthens recognition reliability at the semantic level. However, when used alone, the attention branch yields only limited improvement under stricter overlap thresholds. This pattern suggests that semantic enhancement and geometric precision are not equivalent.

This observation has a clear methodological implication. For geomorphic targets such as landslides, an increase in target saliency does not automatically translate into accurate boundary fitting. A model may become better at recognizing that a certain area is likely to be a landslide, while still failing to place the box tightly around its actual extent. The attention mechanism is therefore necessary, but it cannot serve as a complete solution [32,33,34]. Its real value emerges when it is combined with a structure that can simultaneously preserve geometric fidelity.

### 5.3. Importance of Inner-Scale Interaction

The complete model substantially outperformed the single-branch variants, indicating that the gain of L-SAINet arises not from the isolated presence of the two branches, but from their effective interaction within the same scale. The inner-scale feature interaction mechanism appears to be essential because it allows semantic cues and geometric cues to be aligned within the same representation space [37,38,39,40,41,42].

This design is meaningful for landslide detection because the target is defined by both boundary pattern and contextual distinction. A detector that emphasizes only geometry may better follow local shape but remain vulnerable to confusing background. A detector that emphasizes only semantic saliency may recognize the target region more reliably but still produce loose or biased boxes. When these two types of information interact at the same scale, the boundary response is constrained by target significance, and semantic focus is supported by more accurate spatial structure. This mutual reinforcement explains why the complete framework shows clear advantages in both detection reliability and localization consistency [24,27,32,33,34,35,36,37].

The qualitative examples further support this interpretation. In elongated or fragmented landslides, the full model provides tighter and more complete boxes than the baseline and the single-module variants. In vegetation-covered or shadow-affected scenes, the model remains capable of isolating relevant target regions without obvious over-expansion into surrounding terrain. These behaviors suggest that the interaction mechanism helps stabilize intermediate representation under complex conditions, which is a crucial requirement for geological hazard interpretation.

From a methodological perspective, the proposed design is motivated by the fact that landslide detection requires both boundary-sensitive geometric representation and background-aware semantic discrimination. A geometry-oriented branch alone may improve spatial alignment but remain vulnerable to confusing background regions, whereas a semantics-oriented branch alone may improve target saliency while still producing loose or biased boxes. The rationale of L-SAINet is therefore to couple these two complementary responses within the same feature scale before prediction, so that target extent modeling and background suppression can be jointly refined. In this sense, the contribution of the present study lies in a task-oriented coupling design for irregular geomorphic targets rather than in proposing entirely new basic operators.

### 5.4. Limitations and Future Work

Although the proposed framework achieved encouraging results, several limitations should be acknowledged. First, the present study was conducted on a single optical remote sensing dataset from one representative region. The reported conclusions therefore mainly reflect the characteristics of the Bijie Landslide Remote Sensing Dataset. The generalization of the framework to other geomorphic settings, climatic environments, and acquisition conditions still requires systematic verification.

Second, the current task was defined as single-class bounding-box detection. This formulation is suitable for rapid inventory extraction, but it cannot fully represent the detailed geometry of landslide bodies. In many practical applications, especially those related to hazard zoning or process analysis, more precise delineation is desirable. Future work may extend the framework toward instance segmentation or boundary-aware extraction so that finer spatial information can be retained.

Third, the model was developed for single-temporal optical imagery. Under dense vegetation cover, strong shadow, or weak spectral contrast, the visual separability of some landslides remains limited. In such cases, omission may still occur even when localization quality for detected targets is satisfactory. This limitation suggests that future improvement could benefit from the integration of multi-source or multi-temporal information, including terrain factors, multispectral data, or change-sensitive observations [15,18,19,29,30,31,43,44,45].

Finally, although the present study demonstrates the value of shape-adaptive representation and inner-scale feature interaction, the structural design can still be refined. More compact interaction mechanisms, stronger cross-scene validation, and better treatment of small or weakly expressed failures should be considered in future research. Such improvements would further enhance the applicability of the model in operational landslide inventory mapping and rapid hazard interpretation.

In addition, the present experiments were reported based on a single training pipeline rather than repeated runs with multiple random seeds. Although the recorded convergence behavior indicates stable optimization, future work should further evaluate statistical robustness across repeated experiments.

## 6. Conclusions

This study addressed the problem of landslide detection in high-resolution optical remote sensing imagery under complex mountain conditions. The main challenge lies in the irregular geometry of landslide targets, the large variation in scale, and the strong confusion caused by heterogeneous background elements. To improve both recognition reliability and localization consistency, a task-oriented detection framework named L-SAINet was developed.

The proposed framework introduces a Landslide Shape-Aware and Inner-scale interaction module into a lightweight detection architecture. The module combines a shape-adaptive branch, a channel-spatial attention branch, and an inner-scale feature interaction mechanism. The shape-adaptive branch enhances geometric alignment for irregular landslide bodies. The attention branch improves target saliency and reduces confusing background response. The interaction mechanism allows semantic and geometric information to collaborate at the same feature scale, thereby producing a more effective representation for landslide detection.

Experiments conducted on the Bijie Landslide Remote Sensing Dataset show that the proposed framework improves overall detection performance and achieves better localization under strict overlap conditions. The ablation results indicate that semantic refinement and geometric adaptation contribute in different ways, and that the strongest performance is obtained when the two are integrated through inner-scale interaction. Qualitative analysis further shows that the framework maintains relatively stable behavior in shadowed scenes, vegetation-covered slopes, disturbed road-cut zones, and other challenging environments.

Overall, the results suggest that shape-aware feature learning is beneficial for landslide detection in complex optical remote sensing imagery. The proposed framework provides a useful basis for rapid landslide inventory mapping and related hazard interpretation tasks. Future work should focus on cross-region generalization, finer spatial delineation, and the integration of multi-source observations in order to further improve practical applicability.

## Figures and Tables

**Table 1:** Summary of the landslide detection dataset used in this study.

Item	Value
Total images	4392
Training/Validation/Test	3514/439/439
Total landslide instances	5032
Average bounding-box size	118 × 162 pixels
Spatial resolution	High-resolution optical imagery, 0.8 m
Annotation type	Axis-aligned bounding box
Detection task	Single-class landslide detection

**Table 2:** Quantitative comparison with representative detection models on the test set.

Model	Precision	Recall	mAP@0.5	mAP@0.5:0.95	Params (M)	FLOPs (G)	ms/img	FPS
YOLOv8m Baseline	0.69	0.62	0.646	0.104	11.2	28.4	8.6	116.3
YOLOv10n	0.72	0.65	0.673	0.326	11.8	29.7	9.5	106.4
YOLO11n	0.74	0.71	0.735	0.386	12.3	31.4	10.2	98.7
Faster R-CNN	0.79	0.69	0.714	0.354	11.8	30.6	9.8	102.2
L-SAINet	0.86	0.88	0.865	0.612	13.5	34.2	10.9	91.7

**Table 3:** Quantitative comparison of the baseline detector and enhanced variants on the test set.

Model	Precision	Recall	mAP@0.5	mAP@0.5:0.95
YOLOv8m	0.69	0.62	0.646	0.104
YOLOv8m + CBAM	0.78	0.82	0.760	0.129
YOLOv8m + ADC	0.74	0.75	0.750	0.148
L-SAINet	0.86	0.88	0.865	0.612

**Table 4:** Ablation study of the key components in L-SAINet.

Configuration	CBAM	ADC	Inner-Scale Interaction	Precision	Recall	mAP@0.5	mAP@0.5:0.95
Baseline	×	×	×	0.69	0.62	0.646	0.104
Baseline + CBAM	√	×	×	0.78	0.82	0.760	0.129
Baseline + ADC	×	√	×	0.74	0.75	0.750	0.148
Baseline + CBAM + ADC	√	√	×	0.80	0.83	0.792	0.228
L-SAINet	√	√	√	0.86	0.88	0.865	0.612

**Table 5:** Model complexity and inference efficiency of different configurations.

Model	Parameters (M)	FLOPs (G)	Inference Time per Image (ms)	FPS	mAP@0.5	mAP@0.5:0.95
YOLOv8m Baseline	11.2	28.4	8.6	116.3	0.646	0.104
YOLOv8m + CBAM	11.8	29.6	9.1	109.9	0.760	0.129
YOLOv8m + ADC	12.4	31.8	9.8	102.0	0.750	0.148
YOLOv8m + CBAM + ADC	13.0	33.1	10.4	96.2	0.792	0.228
L-SAINet	13.5	34.2	10.9	91.7	0.865	0.612

**Table 6:** Detection performance under representative complex scene categories.

Scene Category	No. of Test Images	Baseline Recall	L-SAINet Recall	Baseline mAP@0.5	L-SAINet mAP@0.5
Shadow-affected low-contrast scenes	52	0.54	0.79	0.59	0.82
Vegetation-covered slopes	61	0.58	0.81	0.61	0.84
Road-cut disturbance scenes	48	0.63	0.84	0.66	0.86
Small landslides near linear infrastructure	44	0.49	0.74	0.52	0.77
Multi-scale coexisting landslides	57	0.66	0.86	0.69	0.88
Irregular landslides in complex textured backgrounds	63	0.55	0.80	0.58	0.83

## Data Availability

The Bijie Landslide Remote Sensing Dataset used in this study is publicly available on the official data repository of Wuhan University at: https://gpcv.whu.edu.cn/data/data.html (accessed on 1 April 2026). No new data were created or analyzed in this study.

## Abbreviations

The following abbreviations are used in this manuscript:

ADC	Adaptive Deformable Convolution
CBAM	Convolutional Block Attention Module
CNN	Convolutional Neural Network
IoU	Intersection over Union
L-SAI	Landslide Shape-Aware and Inner-scale Interaction
L-SAINet	Landslide Shape-Aware and Inner-scale Interaction Network
mAP	Mean Average Precision
PR	Precision–Recall
RS	Remote Sensing
FLOPs	Floating-Point Operations
FPS	Frames Per Second
